# Herpes simplex virus type 2 inhibits TNF-α-induced NF-κB activation through viral protein ICP22-mediated interaction with p65

**DOI:** 10.3389/fimmu.2022.983502

**Published:** 2022-09-23

**Authors:** Huimin Hu, Ming Fu, Chuntian Li, Binman Zhang, Yuncheng Li, Qinxue Hu, Mudan Zhang

**Affiliations:** ^1^ State Key Laboratory of Virology, Wuhan Institute of Virology, Center for Biosafety Mega-Science, Chinese Academy of Sciences, Wuhan, China; ^2^ Savaid Medical School, University of Chinese Academy of Sciences, Beijing, China; ^3^ Department of Gastroenterology, Guangzhou Women and Children’s Medical Center, Guangzhou Medical University, Guangzhou, China; ^4^ Institute for Infection and Immunity, St George’s, University of London, London, United Kingdom

**Keywords:** HSV-2, ICP22, NF-κB, p65, immune evasion

## Abstract

Herpes simplex virus type 2 (HSV-2) is a prevalent human pathogen and the main cause of genital herpes. After initial infection, HSV-2 can establish lifelong latency within dorsal root ganglia by evading the innate immunity of the host. NF-κB has a crucial role in regulating cell proliferation, inflammation, apoptosis, and immune responses. It is known that inhibition of NF-κB activation by a virus could facilitate it to establish infection in the host. In the current study, we found that HSV-2 inhibited TNF-α-induced activation of NF-κB-responsive promoter in a dose-dependent manner, while UV-inactivated HSV-2 did not have such capability. We further identified the immediate early protein ICP22 of HSV-2 as a vital viral element in inhibiting the activation of NF-κB-responsive promoter. The role of ICP22 was confirmed in human cervical cell line HeLa and primary cervical fibroblasts in the context of HSV-2 infection, showing that ICP22 deficient HSV-2 largely lost the capability in suppressing NF-κB activation. HSV-2 ICP22 was further shown to suppress the activity of TNF receptor-associated factor 2 (TRAF2)-, IκB kinase α (IKK α)-, IKK β-, IKK γ-, or p65-induced activation of NF-κB-responsive promoter. Mechanistically, HSV-2 ICP22 inhibited the phosphorylation and nuclear translocation of p65 by directly interacting with p65, resulting in the blockade of NF-κB activation. Furthermore, ICP22 from several alpha-herpesviruses could also inhibit NF-κB activation, suggesting the significance of ICP22 in herpesvirus immune evasion. Findings in this study highlight the importance of ICP22 in inhibiting NF-κB activation, revealing a novel mechanism by which HSV-2 evades the host antiviral responses.

## Introduction

HSV-2 is a large dsDNA virus, a member of the Herpesviridae family, belonging to genus Simplexvirus. The WHO reported that about 13% of the world’s population aged 15 to 49 years were living with HSV-2 infection in 2016 (1). HSV-2 is mainly sexually transmitted and infects epithelial cells, causing genital herpes (2). It also infects leukocytes and neuronal cells (3–5), leading to encephalitis and disseminated diseases that affect other organ systems (6, 7). After initial infection, HSV-2 can establish life-long latency in the dorsal root ganglia (8).

It is known that HSV (HSV-1/2) has evolved countermeasures to evade the host innate immune responses. However, most of the studies to date have been focusing on HSV-1 (9). For instance, the UL2, UL24, UL42, RL2 and US3 of HSV-1 can interfere with the NF-κB signaling pathway by interacting with p65 or p50, the key component of NF-κB heterodimer (10–14), while HSV-1 UL36 suppresses NF-κB activation by cleaving the polyubiquitin chains of IκB α, an inhibitor of NF-κB activation (15). The RL2, RL1 and UL48 of HSV-1 suppress the production of type I interferons (IFN) by acting on IRF3 (16, 17), whereas the UL54, US11 and UL46 of HSV-1 act on TBK1 to block the production of type I IFN (18–20). In addition, the RL2 and UL41 of HSV-1 were reported to suppress the production of IFN-stimulated genes (ISGs) by degrading IFI16 (21). So far, little is known concerning how HSV-2 evades the host innate immune system. We previously demonstrated that the immediate early protein ICP22 of HSV-2 not only suppresses IFN-β production by blocking the association of IRF-3 with IFN-β promoter (22), but also inhibits the production of ISGs by directly degrading IFN-stimulated gene factor 3 (ISGF3) (23). Although ICP22 is a key mediator of HSV-2 immune evasion in type I IFN production and signaling, it remains to be determined whether HSV-2 ICP22 could inhibit the activation of NF-κB signaling pathway.

TNF receptor-associated factor 2 (TRAF2), IκB kinase (IKK) α (IKK α), IKK β, IKK γ and p65 are key components of the NF-κB signaling pathway. Under foreign stimuli, the IKK complex is first activated, resulting in the phosphorylation of IκB proteins and its subsequent degradation by the proteasome. Once IκB protein is detached, released NF-κB dimers are phosphorylated and then translocate to the nucleus to activate the transcription of target genes (24–26). Given that a number of viruses were previously shown to block the activation of NF-κB by acting on different components of NF-κB signaling pathway (27–33), we asked whether and how HSV-2 inhibits NF-κB activation.

In this study, we first revealed that HSV-2 blocks TNF-α-induced activation of NF-κB-responsive promoter. We further demonstrated that the immediate early protein ICP22 of HSV-2 inhibits NF-κB activation by directly interacting with p65, resulting in the blockade of p65 phosphorylation and nuclear translocation.

## Materials and methods

### Cell lines and viruses

HEK 293T, human cervical epithelial cell line HeLa, and African green monkey kidney cell line Vero were purchased from American Type Culture Collection and cultured in Dulbecco’s modified Eagle medium (DMEM) (Gibico, C11995500BT) supplemented with 10% fetal bovine serum (FBS) (Gibico, 10099-141), and 100 U/mL of penicillin and streptomycin each (Genom, GMN15140) at 37°C in a 5% CO_2_ incubator. Primary human cervical fibroblasts were purchased from Meisen Chinese Tissue Culture Collections (Meisen CTCC, CTCC-088-HUM) (Zhejiang, China) and cultured in primary fibroblast culture medium (Meisen CTCC, CTCC-003-PriMed). HSV-2 (G strain) was obtained from LGC standards and propagated in Vero cells. Virus stock supplemented with 10% FBS (Gibico, 10099-141) was stored at –80°C before being used for infection. UV-inactivated HSV-2 was obtained by exposure to UV irradiation for 30 min. HSV-2 titration was determined by plaque assay on Vero monolayers. ICP22 deficient HSV-2, named *us1* del HSV-2, was constructed and produced as previously described (22).

### Antibodies, reagents and plasmids

Rabbit anti-p65 (10745-1-AP), mouse anti-β-actin (66009-1-Ig) and HA-tag (66006-2-Ig) polyclonal antibodies were purchased from Proteintech (Wuhan, China). Mouse anti-IκB α (L35A5) and phospho-p65 (ser536) (93H1) antibodies were purchased from Cell Signaling Technology. Mouse antibodies against IKK α (sc-7606), IKK β (sc-271782), and IKK γ (sc-8032), respectively, were purchased from Santa Cruz Biotechnology. A mouse anti-Flag monoclonal antibody (mAb) (F1804) was obtained from Sigma-Aldrich. A sheep polyclonal antibody against HSV-2 (ab21112) was purchased from Abcam. Mouse normal IgG (A7028) was purchased from Beyotime. Rabbit normal IgG (A7016) was purchased from Beyotime. Recombinant human TNF-α (300-01A-50) was purchased from PeproTech. HA-tagged plasmids pHA-TRAF2, pHA-IKK α, pHA-IKK β, pHA-IKK γ, and Flag-tagged plasmid pFlag-p65 and the reporter plasmids phRL-TK and pNF-κB-Luc were kindly provided by Professor Hanzhong Wang at the Wuhan Institute of Virology, Chinese Academy of Sciences (34, 35). The coding sequences (CDS) of PRV ICP22 (Gene ID: 2952489) and VZV ICP22 (Gene ID: 1487700) were synthesized and cloned into pcDNA3.1(+) vector, respectively, by GeneCreate Biological Engineering Co, Ltd. (Wuhan, China). The Flag-tagged expression plasmids of HSV-1 ICP22 and HSV-2 UL46 and ICP22 were described in our previous studies (22, 36, 37). All the constructs were verified by DNA sequencing (Sunny Biotechnology, China).

### Dual luciferase reporter assay

HEK 293T cells seeded in 24-well plates overnight were co-transfected with Firefly luciferase reporter plasmid pNF-κB-Luc, Renilla luciferase reporter plasmid phRL-TK and empty vector or plasmid encoding indicated viral protein. Transfections were performed using Lipofectamine 2000 (Invitrogen, 11668-027) according to the manufacturer’s instructions. At 24 h post-transfection, cells were mock-treated or treated with TNF-α (20 ng/ml) for 6 h. In some cases, HEK 293T cells were co-transfected with pNF-κB-Luc, phRL-TK, plasmid expressing pFlag-p65, pHA-TRAF2, pHA-IKK α, pHA-IKK β or pHA-IKK γ and empty vector or plasmid expressing indicated viral protein for 30 h. For HeLa cells, after co-transfection with reporter plasmids pNF-κB-Luc and phRL-TK for 4 h, cells were infected or mock infected with HSV-2, UV-inactivated HSV-2 or *us1* del HSV-2 for 20 h, followed by stimulation with or without TNF-α (20 ng/ml) for 6 h. For primary human cervical fibroblasts, after co-transfection with reporter plasmids pNF-κB-Luc and phRL-TK for 4 h, cells were infected with HSV-2 or *us1* del HSV-2 for 20 h, followed by stimulation with or without TNF-α (20 ng/ml) for 6 h. Cells were subsequently harvested and lysed to measure Firefly and Renilla luciferase activities using a Dual Luciferase Reporter (DLR) Assay System (Promega, E1980) according to the manufacturer’s instructions.

### Western blot

The proteins from transfected or infected cells were prepared using Lysis Buffer supplemented with protease inhibitor cocktail (Roche, 11697498001). Prepared cell lysates or immunoprecipitates were subjected to 10% SDS-PAGE and transferred to 0.45 µm polyvinylidene difluoride (PVDF) membranes (Millipore, IPVH00010 PORE). The membrane was blocked using 5% non-fat milk in TBST (20 mM Tris-HCl buffer [pH 7.4] containing 37 mM NaCl and 0.1% Tween 20) at 4°C for 1 h, followed by incubation with a primary Ab overnight at 4°C. After three washes with TBST, the membrane was probed with a HRP-conjugated secondary Ab (Proteintech, SA00001-1 or SA00001-2) at room temperature for 1 h, and then washed five times with TBST. The bands were visualized by exposure to ChemiDoc MP Imaging System after the addition of chemiluminescent substrate.

### RNA isolation and quantitative PCR

Cells were collected to extract total RNA using TRIzol (Invitrogen, 15596-026) according to the manufacturer’s instructions. Subsequently, cDNA was synthesized with the HiScript II Q RT SuperMix for qPCR (+gDNA wiper) (Vazyme Biotech, R223-01). Relative real-time quantitative PCR was performed on a CFX Real-Time PCR system (Bio-Rad) using ChamQ SYBR qPCR Master Mix (High ROX Premixed) (Vazyme Biotech, Q341-02). The specific primer sequences were as follows: 5′-GCCATTCTGATTTGCTGC-3′ (forward) and 5′-CCTTTCCTTGCTAACTGC-3′ (reverse) for CXCL10, 5′-GGAAATCCCATCACCATC-3′ (forward) and 5′- CATCACGCCACAGTTTCC-3′ (reverse) for GAPDH. The expression difference was calculated on the basis of 2^-ΔΔCt^ values.

### Co-immunoprecipitation assay

HeLa cells seeded in 6-well plates were transfected with Flag-tagged ICP22-expressing plasmid or empty vector. At 24 h post-transfection, cells were mock-treated or treated with TNF-α (20 ng/mL) for 6 h. Cells were subsequently harvested and lysed on ice for 30 min using 200 μL lysis buffer (50 mM Tris [pH 8.0], 150 mM NaCl, 1% NP40) containing protease inhibitor cocktail (Roche, 11697498001). 3 μg mouse anti-Flag Ab or mouse normal IgG (BOSTER, BA1051) was added to fresh Dynabeads protein G (Invitrogen, 10003D), and mixed with cell lysate, respectively. In some cases, 3 μg rabbit anti-p65 Ab or rabbit normal IgG (Beyotime, A7016) was mixed with fresh Dynabeads protein G, and the mixture was then added to cell lysates. After incubation with rotation overnight at 4°C, the Ag-Ab-dynabead complexes were washed three times with PBST, and then the target antigens (Ags) were subjected to western blot analysis after elution followed by boiling.

### Binding kinetic analysis

Human recombinant p65 protein was purchased from SinoBiological Incorporation (12054-H09E, China). HA-tagged ICP22 was purified as described previously (23). Briefly, for every 1 × 10^6^ cells, 1.5 μg expression plasmid was transfected into HEK 293F cells using Polyethylenimine (PEI) transfection reagent (Polysciences, 23966-1, China). Cells were cultured in FreeStyle 293 Expression Medium (Gibico, 12338018, USA) at 37°C in a 5% CO_2_ incubator shaker at 110 rpm. At day 3 post-transfection, cells were harvested and lysed by ultrasonic treatment. The HA-tagged protein was purified by anti-YPYDVPDYA Affinity Resin (DIA•AN, KAP0063, China) and eluted with 300 μg/mL YPYDVPDYA peptide (GenScript, RP11735, China). The purified protein was concentrated in PBS using 10 kDa Centrifugal Filter Units (Merck, UFC901096, Germany) for binding kinetic study.

The kinetics of binding was performed on a Forte-Bio Octet Red System. After Protein A Biosensors (Fortebio, 18-5010, USA) were soaked in 1× PBS, 5 μg/mL anti-p65 Ab was diluted and captured by the Biosensors. The Abs-captured Biosensors were used to bind p65, and then immersed in different concentration of ICP22 (62.5, 125, 250, 500 or 1000 nM) for association and disassociation. The response in nm shift was recorded as a function of time.

### Immunofluorescence analysis

HeLa cells seeded in 35-mm glass-bottom dishes were transfected with Flag-tagged ICP22-expressing plasmid. At 24 h post-transfection, cells were treated with or without 20 ng/mL TNF-α for 6 h, and then fixed with 4% paraformaldehyde at room temperature for 10 min. After permeabilized with 0.2% Triton X-100 at room temperature for 10 min, cells were blocked in PBS containing 3% BSA at 4°C overnight. Thereafter, cells were incubated with the rabbit anti-human p65 polyclonal Ab (pAb) and the mouse anti-Flag mAb for 1 h at 37°C. After three washes with PBS, cells were then incubated with Alexa Fluor 488-labeled Goat anti-Mouse IgG (H+L) (Invitrogen, A-10667) and Alexa Fluor 647-labeled Goat anti-Rabbit IgG (H+L) (Invitrogen, A27018) for 1 h at 37°C. Cells were subsequently washed and incubated with DAPI for 10 min at 37°C. After washes, cells were observed under a fluorescence microscope (Nikon A1R/MP).

### Statistical analysis

All experiments were repeated at least three times and the data were presented as mean ± SD unless otherwise specified. Data analyses were performed with GraphPad Prism 7.0 software (GraphPad). Comparison between two groups was analyzed by Student t-test, whereas comparisons among more than two groups were analyzed by one-way ANOVA with the Tukey’s test. P < 0.05 was considered statistically significant.

## Results

### Productive HSV-2 infection suppresses TNF-α-induced NF-κB activation

**Figure 1:**
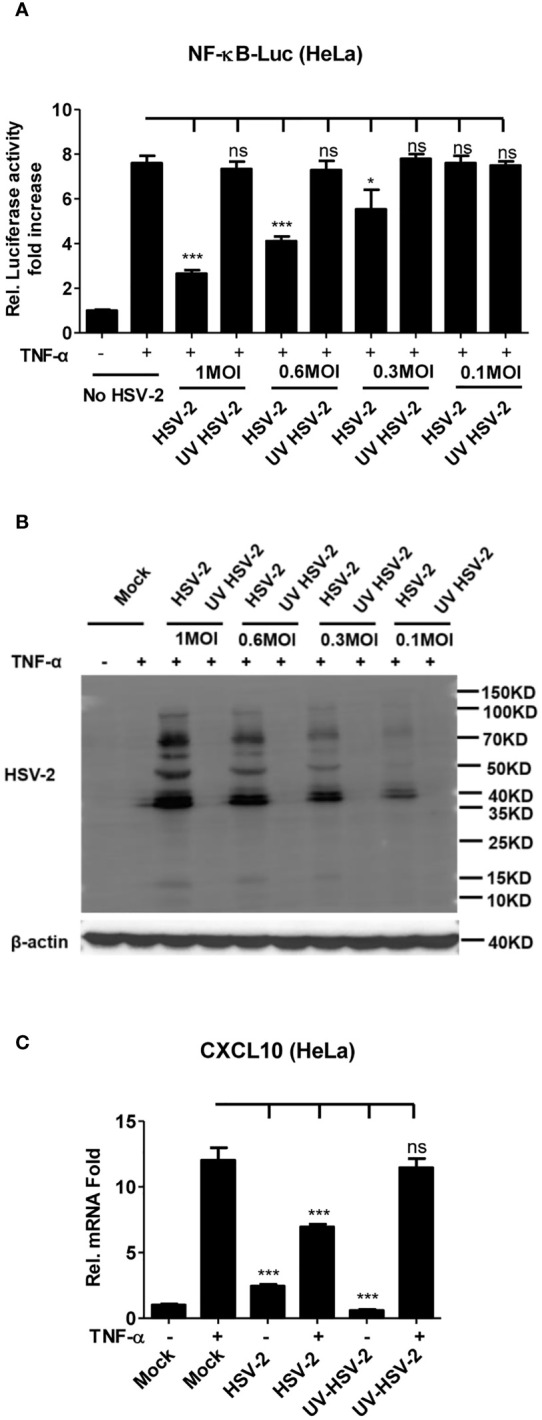
Productive HSV-2 infection suppresses TNF-α-induced NF-κB activation. **(A)** Productive HSV-2 infection suppresses TNF-α-induced NF-κB activation. HeLa cells were seeded in 24-well plates overnight and co-transfected with the reporter plasmids pNF-κB-Luc and phRL-TK. At 4 h post-transfection, cells were mock infected or infected with HSV-2 or UV-inactivated HSV-2 (UV HSV-2) at an MOI of 1, 0.6, 0.3, or 0.1. At 20 h post-infection, cells were stimulated with or without TNF-α (20 ng/ml) for 6 h. Reporter activities were determined by DLR assay. **(B)** Detection of viral protein expression in HSV-2-infected cells. HeLa cells were infected with HSV-2 or UV-inactivated HSV-2 at an MOI of 1, 0.6, 0.3, or 0.1 for 24 h. The expression of viral protein was detected by western blot using the anti-HSV-2 Ab. β-actin was used as a loading control. **(C)** HSV-2 infection inhibits CXCL10 mRNA production. HeLa cells seeded in 6-well plates were infected with HSV-2 or UV-inactivated HSV-2. At 24 h post-infection, cells were stimulated with TNF-α (20 ng/ml) for 6 h. Cells were harvested and total RNA was extracted. The expression of CXCL10 and GAPDH genes was evaluated by relative real-time quantitative PCR. For graphs, data shown are mean ± SD of three independent experiments with each condition performed in triplicate. For images, one representative experiment out of three is shown. *p < 0.05, ***p < 0.001. ns, not significantly. Rel, Relative.

It is known that HSV-2 can evade the host innate immunity to establish lifelong infection. Considering the critical role of NF-κB in the innate immunity, we examined the effect of HSV-2 infection on NF-κB activation. Given that human genital epithelial cells are the main targets of HSV-2 primary infection, we used human cervical epithelial cell line HeLa for the initial experiment. HeLa cells were co-transfected with the reporter plasmids pNF-κB-Luc and phRL-TK for 4 h, followed by infection with HSV-2 or UV-inactivated HSV-2 at an MOI of 1, 0.6, 0.3, or 0.1 for 20 h. After 6 h stimulation with TNF-α, the activities of luciferase were detected. As shown in **Figure 1A**, HSV-2 significantly inhibited the activation of NF-κB-responsive promoter, whereas UV-inactivated HSV-2 did not have such capability. We further confirmed that the viral proteins were barely detectable by WB after HSV-2 was inactivated by UV (**Figure 1B**), indicating that productive infection is necessary for HSV-2-mediated inhibition of NF-κB activation. Given that the chemokine CXCL10 could be induced *via* NF-κB activation (38, 39), we detected whether HSV-2 infection affects CXCL10 mRNA production. As showed in **Figure 1C**, HSV-2 infection indeed inhibited NF-κB activation-induced CXCL10 mRNA production, further suggesting the inhibitory effect of HSV-2 on NF-κB activation. These results indicate that HSV-2 infection suppresses TNF-α-induced NF-κB activation and that productive HSV-2 infection is necessary for such suppression.

### HSV-2 ICP22 inhibits TNF-α-induced NF-κB activation

**Figure 2:**
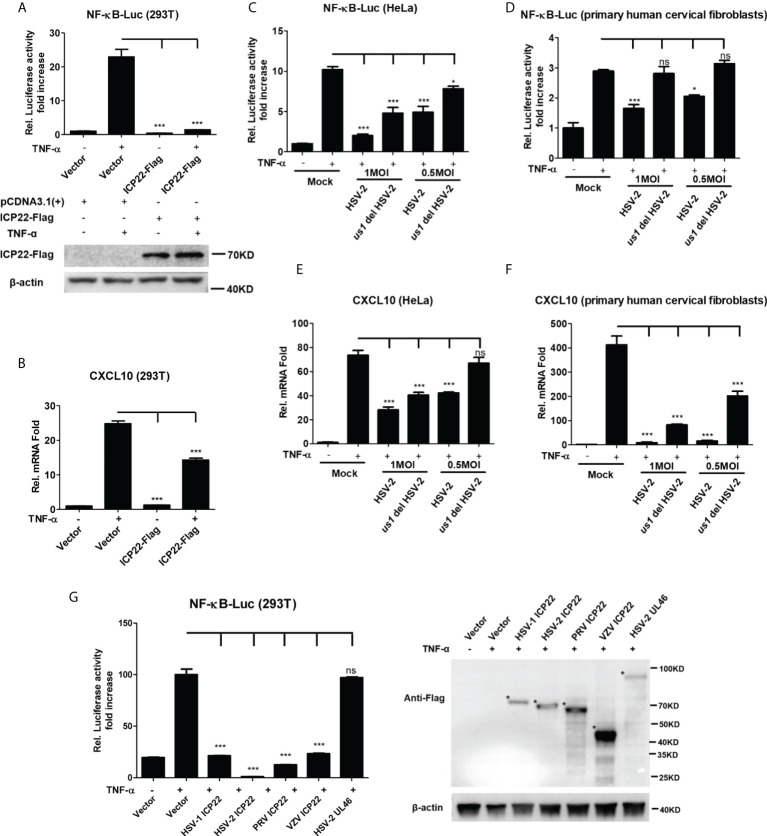
HSV-2 ICP22 inhibits TNF-α-induced NF-κB activation. **(A)** HSV-2 ICP22 suppresses TNF-α-induced NF-κB activation. HEK 293T cells were seeded in 24-well plates overnight and co-transfected with the reporter plasmids pNF-κB-Luc and phRL-TK together with ICP22-expressing plasmid. At 24 h post-transfection, cells were stimulated with TNF-α (20 ng/ml) for 6 h. Reporter activities were determined by DLR assay. The expression of ICP22 was detected by western blot using the anti-Flag Ab. **(B)** HSV-2 ICP22 suppresses the production of CXCL10 mRNA. HEK 293T cells seeded in 6-well plates were transfected with vector or ICP22-expressing plasmid. At 24 h post-transfection, cells were stimulated with TNF-α (20 ng/ml) for 6 h. Cells were harvested and total RNA was extracted. The expression of CXCL10 and GAPDH genes was evaluated by relative real-time quantitative PCR. **(C, D)**. ICP22 knockout impairs the inhibitory activity of HSV-2 on NF-κB activation. HeLa cells or primary human cervical fibroblasts were seeded in 24-well plates overnight and transfected with the reporter plasmids pNF-κB-Luc and phRL-TK, followed by infection with HSV-2 or *us1* del HSV-2. After stimulation with TNF-α for 6 h, reporter activities were determined by DLR assay. **(E, F)**. HSV-2 ICP22 knockout impairs the inhibitory activity of HSV-2 on CXCL10 mRNA production. HeLa cells or primary human cervical fibroblasts seeded in 6-well plates were infected with HSV-2 or *us1* del HSV-2 at an MOI of 1. After stimulation with TNF-α for 6 h, cells were harvested and total RNA was extracted. The expression of CXCL10 and GAPDH genes was evaluated by relative real-time quantitative PCR. **(G)** ICP22s from several alpha-herpesviruses significantly inhibit NF-κB activation. HEK 293T cells were co-transfected with the reporter plasmids pNF-κB-Luc and phRL-TK together with ICP22-expressing plasmid of HSV-1, PRV or VZV, or expression plasmid of HSV-2 UL46. At 24 h post-transfection, cells were stimulated with TNF-α (20 ng/ml) for 6 h. Reporter activities were determined by DLR assay. The expressions of HSV-1 ICP22-Flag, HSV-2 ICP22-Flag, PRV ICP22-Flag, VZV ICP22-Flag and HSV-2 UL46-Flag were detected by western blot. Asterisk indicated the locations of proteins. For graphs, data shown are mean ± SD of three independent experiments with each condition performed in triplicate. For images, one representative experiment out of three is shown. *p < 0.05, ***p<0.001, ns, not significantly. Rel, Relative.

Our previous studies show that the HSV-2 ICP22 not only suppresses IFN-β production by blocking the association of IRF-3 with IFN-β promoter (22), but also inhibits the production of ISGs by directly degrading ISGF3 (23). Considering the key role of ICP22 in HSV-2-mediated immune evasion, we next assessed the involvement of HSV-2 ICP22 in interfering with NF-κB signaling pathway. HEK 293T cells were co-transfected with the reporter plasmids pNF-κB-Luc and phRL-TK together with ICP22-expressing plasmid. At 24 h post-transfection, cells were stimulated with TNF-α for 6 h. As shown in **Figure 2A**, HSV-2 ICP22 significantly inhibited the activation of NF-κB-responsive promoter. Moreover, HSV-2 ICP22 also suppressed CXCL10 mRNA production (**Figure 2B**). To confirm the role of ICP22 in the inhibition of NF-κB activation in the context of virus infection, HeLa cells or primary human cervical fibroblasts were transfected with the reporter plasmids pNF-κB-Luc and phRL-TK for 4 h, followed by infection with HSV-2 or *us1* del HSV-2, which was constructed as described previously (22), for 20 h, and a stimulation with TNF-α for another 6 h. The results showed that ICP22 knockout significantly impaired the capability of HSV-2 in inhibiting NF-κB activation (**Figures 2C, D**) and CXCL10 mRNA production (**Figures 2E, F**) in both HeLa cells and primary human cervical fibroblasts. To address whether the inhibitory effect of HSV-2 ICP22 on NF-κB activation was virus specific, we assessed the effects of the ICP22s from several alpha-herpesviruses on NF-κB activation. The results showed that the ICP22s of alpha-herpesviruses HSV-1, PRV and VZV all significantly inhibited NF-κB activation, whereas HSV-2 UL46 had no such effect (**Figure 2G**), suggesting the significance of ICP22 in herpesvirus immune evasion. These results collectively indicate that HSV-2 ICP22 inhibits TNF-α-induced NF-κB activation.

### HSV-2 ICP22 inhibits NF-κB activation by acting on the downstream of p65

**Figure 3:**
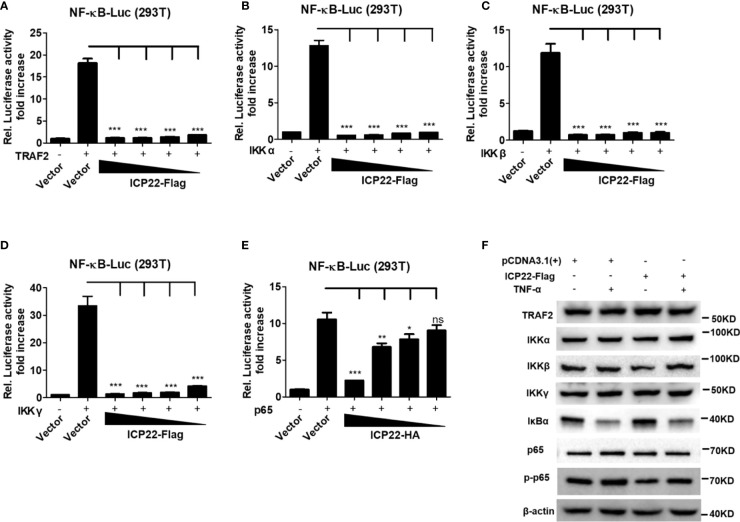
HSV-2 ICP22 inhibits NF-κB activation by acting on the downstream of p65. **(A–E)**. HSV-2 ICP22 inhibits TRAF2, IKK α, IKK β, IKK γ and p65-induced NF-κB activation. HEK 293T cells were seeded in 24 well plates overnight and co-transfected with the reporter plasmids pNF-κB-Luc and phRL-TK, and plasmid expressing TRAF2, IKK α, IKK β, IKK γ or p65, together with empty vector or ICP22-expressing plasmid. At 30 h post-transfection, the reporter activities were determined by DLR assay. **(F)**. HSV-2 ICP22 has no effect on the expression of TRAF2, IKK α, IKK β, IKK γ and p65 or degradation of IκB α. HEK 293T cells were seeded in 6 well plates overnight and transfected with plasmid expressing ICP22 or empty vector. At 24 h post-transfection, cells were stimulated with or without TNF-α (20 ng/ml) for 6 h. The expressions of TRAF2, IKK α, IKK β, IKK γ, IκB α, p65 and phospho-p65 were detected by western blot. For graphs, data shown are mean ± SD of three independent experiments with each condition performed in triplicate. For images, one representative experiment out of three is shown. *p<0.05, **p<0.01, ***p<0.001, ns, not significantly.

IκB protein inhibits the activation of NF-κB by trapping NF-κB in the cytoplasm (40). Under foreigner stimuli, IKK complex phosphorylates IκB, resulting in the phosphorylation, and subsequent degradation of IκB. Once IκB is detached from NF-κB, NF-κB is phosphorylated and activated (41). To understand how HSV-2 ICP22 antagonizes NF-κB activation, HEK 293T cells were co-transfected with the reporter plasmids pNF-κB-Luc and phRL-TK, and the plasmid expressing TRAF2, IKK α, IKK β, IKK γ or p65, together with ICP22-expressing plasmid or empty vector for 30 h. As showed in **Figures 3A–E**, overexpression of TRAF2, IKK α, IKK β, IKK γ or p65 resulted in the activation of NF-κB-responsive promoter, whereas HSV-2 ICP22 significantly inhibited TRAF2, IKK α, IKK β, IKK γ or p65-induced NF-κB activation, without affecting the expression of TRAF2, IKK α, IKK β, IKK γ, and p65 (**Figure 3F**). As showed in **Figure 3F**, in the condition of TNF-α stimulation, the inhibitory factor IκB α was degraded in both vector- and ICP22-transfected cells indicating that HSV-2 ICP22 did not affect the degradation of IκB α, while the total level of phospho-p65 was decreased in ICP22-tranfected cells. These results collectively indicate that HSV-2 ICP22 inhibits the activation of NF-κB by acting on the downstream of p65.

### HSV-2 ICP22 inhibits the phosphorylation and nuclear translocation of p65

**Figure 4:**
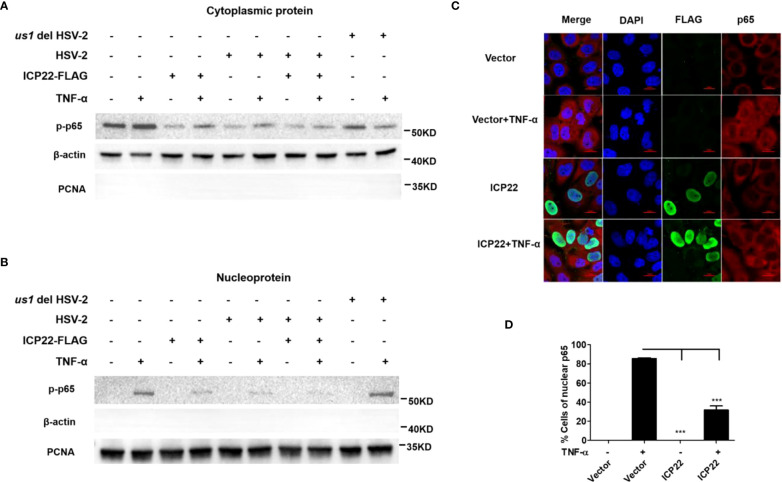
HSV-2 ICP22 inhibits the phosphorylation and nuclear translocation of p65. **(A, B)**. HSV-2 ICP22 inhibits the phosphorylation of p65. HeLa cells seeded in the 6 well plates were transfected with empty vector or ICP22-expressing plasmid. At 4 h post-transfection, cells were mock infected or infected with HSV-2 or *us1* del HSV-2 at an MOI of 1. At 20 h post-infection, cells were stimulated with or without TNF-α (20 ng/ml) for 6 h. The phosphorylated p65 in the cytoplasm and nucleus were detected by western blot. β-actin and PCNA were used as loading controls for cytoplasmic and nuclear proteins, respectively. **(C, D)**. HSV-2 ICP22 significantly inhibits the nuclear translocation of p65. HeLa cells were transfected with ICP22-expressing plasmid or empty vector. At 24 h post-transfection, cells were stimulated with or without TNF-α (20 ng/ml) for 6 h. Cells were stained using the mouse anti-Flag and the rabbit anti-p65 Ab. Alexa Fluor 488-conjugated goat anti-mouse (green) and Alexa Fluor 647-conjugated goat anti-rabbit (red) were used as secondary antibodies. Cell nuclei (blue) were stained with DAPI. The images were obtained by fluorescence microscopy using a 60× objective. The percentage of p65-positive nuclei was quantified in a number of fields **(D)**. The scale bar indicates 20 μm. For graphs, data shown are mean ± SD of three independent experiments with each condition performed in triplicate. For images, one representative experiment out of three is shown. ***p < 0.001.

Given that HSV-2 ICP22 inhibits the activation of NF-κB by acting on the downstream of p65, we next investigated the influence of ICP22 on p65 phosphorylation and nuclear translocation. HeLa cells were transfected with ICP22-expressing plasmid, followed by stimulation with TNF-α for 6 h. Cytoplasmic and nuclear proteins were subsequently isolated and detected by western blot to determine the distribution of p65. As showed in **Figure 4A** (Lane 1-4), the phosphorylation of p65 in the cytoplasm was inhibited by ICP22, although the expression of total p65 was not affected by ICP22 (**Figure 3F**). Meanwhile the phosphorylated p65 in the nucleus also decreased in ICP22-transfected cells (**Figure 4B** Lane 1-4). To further confirm the effect of ICP22 on p65 phosphorylation and nuclear translocation in the context of virus infection, HeLa cells were mock infected or infected with HSV-2 or *us1* del HSV-2. At 24 h post-infection, cells were stimulated with or without TNF-α for 6 h. As showed in **Figures 4A, B**, HSV-2 infection could inhibit the phosphorylation and nuclear translocation of p65 (Lane 5-6), whereas ICP22 knockout obviously impaired the inhibitory effect of HSV-2 on p65 phosphorylation and nuclear translocation (Lane 9-10). Immunofluorescence assay further showed that p65 translocated from the cytoplasm into the nucleus in the majority of cells after stimulation with TNF-α, and such translocation was significantly blocked in ICP22-transfected cells (**Figures 4C, D**). These results together indicate that HSV-2 ICP22 inhibits the phosphorylation and nuclear translocation of p65.

### HSV-2 ICP22 directly interacts with p65

**Figure 5:**
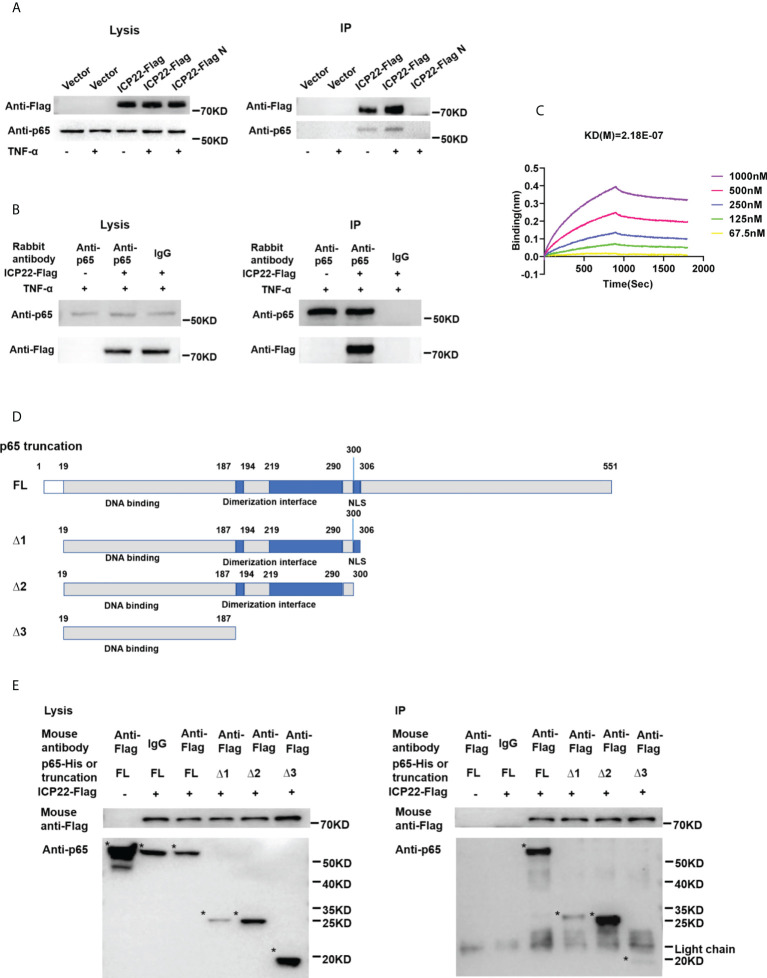
HSV-2 ICP22 directly interacts with p65. **(A)** HSV-2 ICP22 interacts with endogenous p65. **(B)** Endogenous p65 interacts with HSV-2 ICP22. HeLa cells seeded in the 6 well plates were transfected with empty vector or ICP22-expressing plasmid. At 24 h post-transfection, cells were mock-treated or treated with TNF-α (20 ng/ml) for 6 h. Cell lysates were then subjected to co-immunoprecipitation assays using the anti-Flag **(A)** or anti-p65 Ab **(B)**. The mouse **(A)** or rabbit **(B)** non-specific antibody was used as negative control. ICP22 and p65 were detected by western blot using the anti-Flag or anti-p65 Ab, respectively. **(C)** HSV-2 ICP22 directly interacts with p65. The kinetics of binding was performed on a Forte-Bio Octet Red System. 5 μg/mL rabbit anti-p65 Ab was coupled to Protein A biosensors. 25 μg/mL recombinant p65 was bound to Biosenors and immersed in different concentration of ICP22 (62.5, 125, 250, 500 or 1000 nM) for association and disassociation. The response in nm shift was recorded as a function of time. KD (M) = 2.18E-07. **(D)** Schematic representation of p65 truncations. **(E)** HSV-2 ICP22 interacts with the three truncation mutants Δ1, Δ2 and Δ3 of p65. Empty vector or ICP22-expressing plasmid and plasmid expressing full-length or truncated p65 were co-transfected into HEK 293T cells. At 24 h post-transfection, cells were mock-treated or treated with TNF-α (20 ng/ml) for 6 h. Cell lysates were then subjected to co-immunoprecipitation assays using the anti-Flag mAb. ICP22, truncated p65 were detected by western blot using the anti-Flag or anti-p65 Ab, respectively. Asterisk indicated the locations of proteins. One representative experiment out of three is shown.

To address how ICP22 suppresses the phosphorylation of p65, co-immunoprecipitation assay was performed to assess the interaction of HSV-2 ICP22 with p65. As showed in **Figures 5A, B**, HSV-2 ICP22 was found to interact with endogenous p65 in both pull-down experiments using the anti-Flag or anti-p65 antibody. To further confirm the results, recombinant HSV-2 ICP22 and human p65 were used to measure the binding kinetics of ICP22 with p65, showing that HSV-2 ICP22 indeed directly interacts with p65 (**Figure 5C**). p65 contains a conserved Rel homology domain (RHD) at the N terminus, which is responsible for nuclear localization, dimerization and DNA binding (24). To map the functional region of p65 interacting with ICP22, we constructed three truncation mutants Δ1, Δ2 and Δ3 (36). As showed in **Figure 5D**, Δ1 (19-306aa) retains complete RHD of p65, while Δ2 (19-300aa) is deficient in the nuclear localization signal (NLS) domain of RHD. Δ3 (19-187aa) only retains the DNA binding domain of RHD. Subsequently, His-tagged full-length p65 or its truncated mutants (named Δ1, Δ2, and Δ3) were used to identify the functional domain of p65 interacting with HSV-2 ICP22. Co-immunoprecipitation assay showed that full-length p65 and its three truncation mutants Δ1, Δ2 and Δ3 all interacted with ICP22 (**Figure 5E**). The interaction of ICP22 with p65 seemed to be weakened when the dimerization domain of p65 was deleted, indicating that the dimerization domain of p65 likely plays a more important role in the interaction. These results together inform that HSV-2 ICP22 directly interacts with p65, resulting in the blockade of p65 phosphorylation and nuclear translocation.

## Discussion

HSV-2 is one of the most common sexually transmitted viruses worldwide, causing neonatal herpes and genital ulcer disease (42). HSV-2 and HSV-1 are closely related but exhibit substantial differences in latency and reactivation patterns (10–14, 43). NF-κB is a key regulator of a broad range of cellular responses, involved in the induction of inflammation (24, 44–46). Although a number of studies report that HSV-1 has evolved multiple countermeasures to subvert the activation of NF-κB signaling pathway (10–12, 15, 16, 20, 47), our current understanding of HSV-2 immune evasion against the activation of NF-κB is limited.

In this study, we found that HSV-2 infection inhibited TNF-α-induced activation of NF-κB-responsive promoter, whereas UV-inactivated HSV-2 did not have such inhibition, indicating that productive HSV-2 infection is necessary for the inhibition of NF-κB activation. Subsequent studies indicated that ICP22 has a significant inhibitory effect on the activation of NF-κB-responsive promoter, which was further confirmed in the context of viral infection using ICP22 deficient HSV-2. Moreover, we found that the ICP22s from several alpha-herpesviruses including HSV-1, PRV and VZV all inhibited NF-κB activation, which share 62%, 33% and 31% identity, respectively, with the amino acid sequence of HSV-2 ICP22, highlighting the significance of ICP22 in herpesvirus immune evasion. Given that HSV-2 ICP22 can also suppress the production of type I IFN and ISGs (22, 23), our findings collectively informed that ICP22 is a key viral element counteracting not only type I IFN production and signaling but also NF-κB activation.

It is known that NF-κB activation is an attractive target for common human viral pathogens to evade host antiviral responses (34, 48–50). The activation of NF-κB signaling cascade includes the phosphorylation and nuclear translocation of p65, a major component of NF-κB heterodimer. We found that HSV-2 ICP22 significantly blocked TRAF2, IKK α, IKK β, IKK γ and p65-induced NF-κB activation, but did not affect the expression of TRAF2, IKK α, IKK β, IKK γ and p65 or the degradation of IκB α, indicating that HSV-2 ICP22 likely affects p65 activation. We previously demonstrated that HSV-2 ICP22 functions as a novel E3 ubiquitin protein ligase to degrade ISGF3, resulting in the inhibition of type I IFN signaling (23). However, in the current study, HSV-2 ICP22 appears to inhibit NF-κB activation independent of its E3 ubiquitin protein ligase activity, and instead, it suppresses the phosphorylation and nuclear translocation of p65, leading to the inhibition of NF-κB activation.

Mechanistically, we found that HSV-2 ICP22 directly interacts with endogenous p65. In accordance with previous findings, several other viral proteins of HSV-1 have also been shown to interact with p65 (10–14).The NF-κB family shares the RHD at the N-terminus, which contains 300 amino acids and has three functions: sequence specific DNA-binding, dimerization and inhibitory protein binding (24, 41). By assessing three truncated p65, we found that full-length p65 and its three truncation mutants Δ1, Δ2 and Δ3 all interacted with ICP22. The p65 truncation mutant Δ3 (19-187aa) only retains the DNA binding domain of RHD. In agreement, our previous study showed that HSV-2 ICP22 also interacts with the DNA binding domain of IRF-3 (22) to suppress IFN-β production, which indirectly supports our co-immunoprecipitation results in this study. An interaction of ICP22 with the DNA binding domain of p65 likely blocks its association with phosphorylase, resulting in the suppression of p65 phosphorylation and nuclear translocation. Given that HSV-2 ICP22 interacts with the DNA binding domain of p65, it likely facilitates viral immune evasion by interfering with the binding of NF-κB with the promoters of regulatory genes in the nucleus.

It is known that HSV-1 ICP22 is an immediate-early protein and a multifunctional viral regulator. HSV-1 ICP22 not only interacts with RNA polymerase II (51–57) and P-TEFb (58) to regulate viral replication (59), but is also involved in posttranslational modification as viral protein kinases (60–63). To date, little is known about the functions of HSV-2 ICP22. We previously revealed that HSV-2 ICP22 functions as a novel E3 ubiquitin protein ligase to degrade ISGF3 (23) and a key viral element contributing to HSV-2 immune evasion (22). Although beyond the scope of this study, future work is warranted to explore the structural characteristics of HSV-2 ICP22 for its multiple functions.

**Figure 6:**
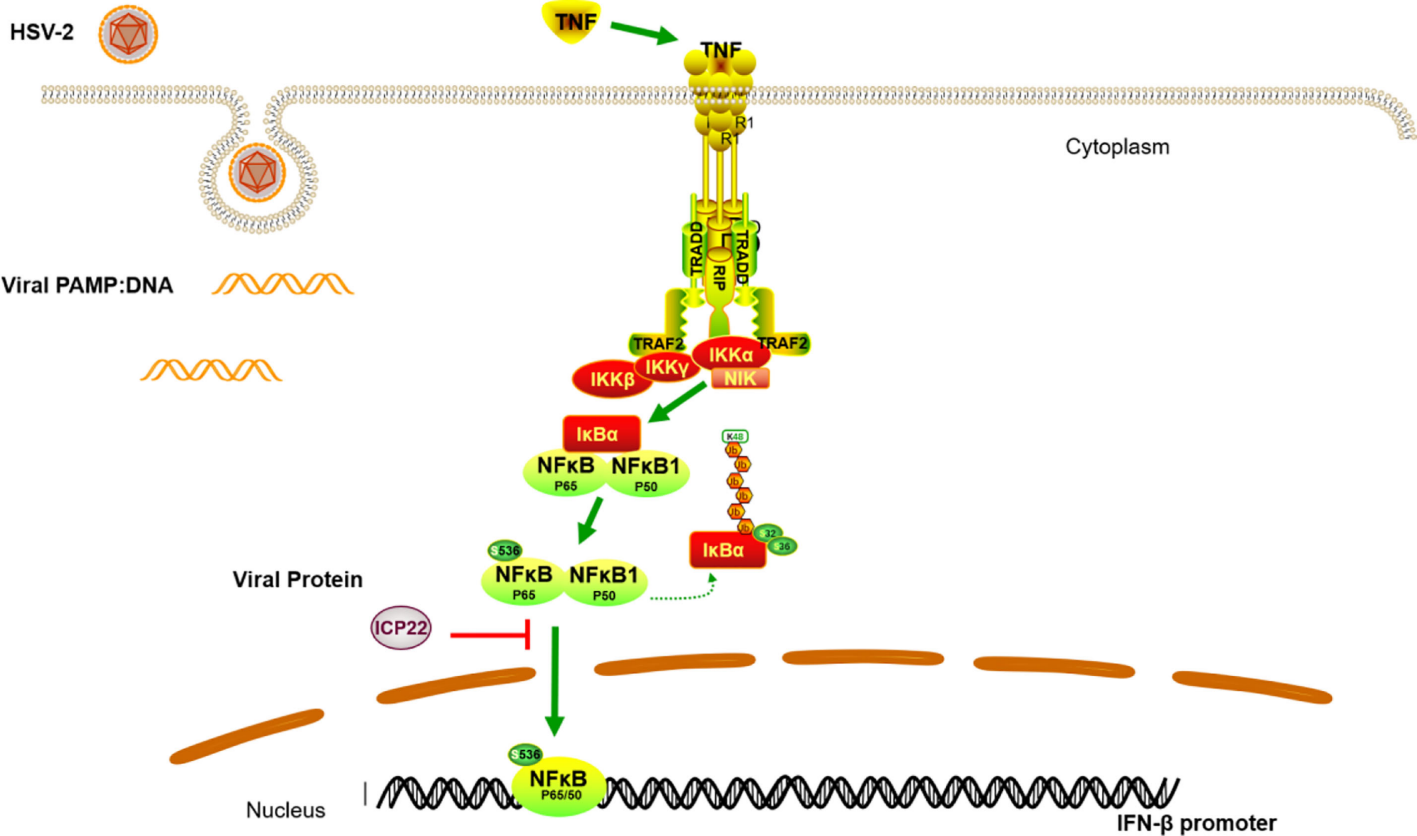
A schematic model of the mechanism by which HSV-2 ICP22 blocks TNF-α-induced NF-κB activation. The host recognizes foreign PAMPs and activates the innate immune system to secret cytokines such as TNF-α. TNF-α subsequently binds TNF receptor (TNFR), resulting in the activation of the IKK complex. The inhibitory protein of NF-κB, IκB α, is then phosphorylated, degraded and detached from NF-κB. NF-κB dimers are then released and phosphorylated, and subsequently translocate to the nucleus to activate the expression of immunomodulatory genes. In the case of HSV-2 infection, the viral immediate early protein ICP22 directly interacts with p65 to block p65 phosphorylation and nuclear translocation, leading to an inhibition of NF-κB activation.

In conclusion, we demonstrated that HSV-2 ICP22 blocks TNF-α-induced activation of NF-κB by directly interacting with p65. The findings highlight the significance of ICP22 in inhibiting NF-κB activation. We proposed a model as described in **Figure 6**. When the host recognizes foreign PAMPs, the innate immune system is activated by secreting cytokines such as TNF-α. The secreted TNF-α engages TNF receptor (TNFR), resulting in the activation of the IKK complex. Subsequently, the inhibitory protein of NF-κB, IκB α, is phosphorylated, degraded and detached from NF-κB. NF-κB dimers are then released and phosphorylated, and subsequently translocate to the nucleus to activate the expression of immunomodulatory genes. In the case of HSV-2 infection, the viral immediate early protein ICP22 directly interacts with p65 to suppress p65 phosphorylation and nuclear translocation, leading to the blockade of NF-κB activation, which would facilitate viral immune escape.

## Data availability statement

The raw data supporting the conclusions of this article will be made available by the authors, without undue reservation.

## Author contributions

HH, MZ and QH conceived the study. HH and MZ conducted most experiments. CL constructed the truncation mutants of p65. MF, BZ and YL provided help in western blot experiments. HH, MZ and QH analyzed the data. HH, MZ and QH wrote the manuscript. All authors contributed to the article and approved the submitted version.

## Funding

This work was supported by National Natural Science Foundation of China (82171736, 81772192 and 31970172), and the National Mega-Projects against Infectious Diseases (2018ZX10301406-002).

## Acknowledgments

We thank Ding Gao at the Center for Instrumental Analysis and Metrology, Wuhan Institute of Virology, Chinese Academy of Sciences for technical assistance of Confocal Microscopy and BioLayer Interferometry.

## Conflict of interest

The authors declare that the research was conducted in the absence of any commercial or financial relationships that could be construed as a potential conflict of interest.

## Publisher’s note

All claims expressed in this article are solely those of the authors and do not necessarily represent those of their affiliated organizations, or those of the publisher, the editors and the reviewers. Any product that may be evaluated in this article, or claim that may be made by its manufacturer, is not guaranteed or endorsed by the publisher.

## Abbreviations

NF-κB, Nuclear factor κB; HIV, Human immunodeficiency virus; HSV, Herpes simplex virus; ICP22, Infected cell protein 22; IFNs, Interferons; IRF3, IFN-regulatory factor 3; TNF-α, Tumor necrosis factor alpha; TNFR, Tumor necrosis factor receptor; TRAF2, TNFR-associated factor 2; IKK, Inhibitor κB (IκB) kinase complex; IKK α, IκB kinase (IKK) α; ISGs, Interferon-stimulated genes; ISGF3, IFN-stimulated gene factor 3; RHD, Rel homology domain; NLS, Nuclear localization sequence.
